# Tris(3-amino-5,6-dimethyl-1,2,4-triazine-κ*N* ^2^)silver(I) trifluromethane­sulfonate–3-amino-5,6-dimethyl-1,2,4-triazine (1/1)

**DOI:** 10.1107/S1600536811040748

**Published:** 2011-10-12

**Authors:** Yu-Hang Jiang, Li-Na Cui, Xu Huang, Qiong-Hua Jin, Cun-Lin Zhang

**Affiliations:** a Department of Chemistry, Capital Normal University, Beijing 100048, People’s Republic of China; b Key Laboratory of Terahertz Optoelectronics, Ministry of Education, Department of Physics, Capital Normal University, Beijing 100048, People’s Republic of China

## Abstract

The asymmetric unit of the title compound, [Ag(C_5_H_8_N_4_)_3_](CF_3_O_3_S)·C_5_H_8_N_4_, contains two cations, two anions and two uncoordinated 3-amino-5,6-dimethyl-1,2,4-triazine (admt) ligands. It was prepared from the reaction of silver trifluoro­methane­sulfonate and admt in a 2:3 molar ratio. Both silver(I) ions are bonded to three admt mol­ecules *via* their 2-position triazine N atoms in almost regular trigonal–planar geometries. Three intra­molecular N—H⋯N hydrogen bonds between adjacent admt mol­ecules in each cation help to maintain their overall near planarities (r.m.s. deviations for the 28 non-H atoms = 0.139 and 0.153 Å). In the crystal, numerous N—H⋯N, N—H⋯O, C—H⋯O, C—H⋯N and C—H⋯F hydrogen-bonding interactions link the components into a three-dimensional network.

## Related literature

For background to the properties and applications of silver complexes, see: Jin *et al.* (2010*a* ▶,*b* ▶); Effendy *et al.* (2007 ▶). Sang & Xu (2006 ▶). For related structures, see: Self *et al.* (1991 ▶); Wang & Cheng (2007 ▶); Liu *et al.* (2002 ▶); Jiang *et al.* (2011 ▶).
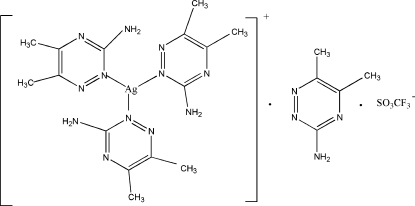


## Experimental

### 

#### Crystal data

[Ag(C_5_H_8_N_4_)_3_](CF_3_O_3_S)·C_5_H_8_N_4_
*M* *_r_* = 753.56
Triclinic, 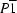

*a* = 13.9357 (14) Å
*b* = 15.1693 (15) Å
*c* = 16.2257 (17) Å
α = 76.735 (1)°
β = 72.565 (1)°
γ = 81.201 (2)°
*V* = 3172.0 (6) Å^3^
*Z* = 4
Mo *K*α radiation
μ = 0.77 mm^−1^
*T* = 298 K
0.45 × 0.27 × 0.23 mm

#### Data collection

Bruker SMART CCD diffractometer
Absorption correction: multi-scan (*SADABS*; Bruker, 2007 ▶) *T*_min_ = 0.723, *T*_max_ = 0.843
16692 measured reflections
11051 independent reflections
4939 reflections with *I* > 2σ(*I*)
*R*_int_ = 0.051

#### Refinement

*R*[*F* ^2^ > 2σ(*F* ^2^)] = 0.069
*wR*(*F* ^2^) = 0.192
*S* = 1.05
11051 reflections
827 parameters
H-atom parameters constrained
Δρ_max_ = 0.91 e Å^−3^
Δρ_min_ = −0.62 e Å^−3^

### 

Data collection: *SMART* (Bruker, 2007 ▶); cell refinement: *SAINT-Plus* (Bruker, 2007 ▶); data reduction: *SAINT-Plus*; program(s) used to solve structure: *SHELXS97* (Sheldrick, 2008 ▶); program(s) used to refine structure: *SHELXL97* (Sheldrick, 2008 ▶); molecular graphics: *SHELXTL* (Sheldrick, 2008 ▶); software used to prepare material for publication: *SHELXTL*.

## Supplementary Material

Crystal structure: contains datablock(s) global, I. DOI: 10.1107/S1600536811040748/hb6434sup1.cif

Structure factors: contains datablock(s) I. DOI: 10.1107/S1600536811040748/hb6434Isup2.hkl

Additional supplementary materials:  crystallographic information; 3D view; checkCIF report

## Figures and Tables

**Table:** 

Ag1—N10	2.241 (6)
Ag1—N6	2.248 (6)
Ag1—N2	2.287 (7)
Ag2—N14	2.251 (7)
Ag2—N18	2.264 (6)
Ag2—N22	2.273 (6)

**Table:** 

N10—Ag1—N6	121.8 (3)
N10—Ag1—N2	119.6 (3)
N6—Ag1—N2	118.6 (2)
N14—Ag2—N18	119.9 (3)
N14—Ag2—N22	119.5 (3)
N18—Ag2—N22	120.0 (2)

**Table 2:** Hydrogen-bond geometry (Å, °)

*D*—H⋯*A*	*D*—H	H⋯*A*	*D*⋯*A*	*D*—H⋯*A*
N4—H4*A*⋯N5	0.86	2.15	2.977 (9)	161
N8—H8*A*⋯N9	0.86	2.22	3.077 (10)	173
N12—H12*B*⋯N1	0.86	2.17	3.018 (11)	169
N16—H16*B*⋯N17	0.86	2.21	3.052 (10)	165
N20—H20*A*⋯N21	0.86	2.18	3.004 (9)	161
N24—H24*B*⋯N13	0.86	2.19	3.044 (11)	169
N4—H4*B*⋯N30^i^	0.86	2.10	2.935 (11)	162
N8—H8*B*⋯O1^ii^	0.86	2.19	3.032 (11)	168
N12—H12*A*⋯O6^iii^	0.86	2.26	3.047 (11)	152
N16—H16*A*⋯O3^iv^	0.86	2.23	3.032 (11)	155
N20—H20*B*⋯N26^ii^	0.86	2.15	3.003 (9)	172
N24—H24*A*⋯O4	0.86	2.26	3.121 (12)	175
N28—H28*A*⋯N19^ii^	0.86	2.24	3.084 (10)	167
N28—H28*B*⋯O5	0.86	2.22	2.978 (15)	146
N32—H32*A*⋯O2^ii^	0.86	2.17	2.982 (13)	156
N32—H32*B*⋯N3^i^	0.86	2.30	3.142 (12)	166
C5—H5*B*⋯O4	0.96	2.56	3.486 (17)	161
C9—H9*A*⋯N27^v^	0.96	2.56	3.363 (12)	141
C15—H15*B*⋯O3^iv^	0.96	2.58	3.538 (15)	177
C35—H35*C*⋯F1^iv^	0.96	2.54	3.375 (14)	145
C40—H40*A*⋯F5^iii^	0.96	2.43	3.208 (17)	138

Symmetry codes: (i) 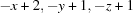; (ii) 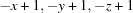; (iii) 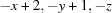; (iv) 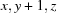; (v) 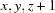.

**Fig. 1.**
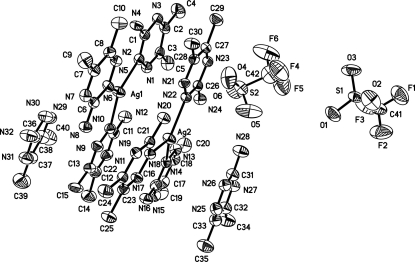
Perspective view of a basic unit of the title complex. Atoms are displayed as elliposoids at the 50% probability level

## supplementary crystallographic information

### Comment

Papers on structural and kinetic features of silver(I) complexes containing heterocyclic-N ligands are growing explosively due to their participation in biological process and their applicatioins in lunminescence and catalysis materials (Jin *et al.*,2010*a*, 2010*b*, Effendy *et al.*, 2007, Sang *et al.*,2006). In our research, we select multifunctional ligand 3-Amino-5,6-dimethyl- 1,2,4-triazine (ADMT) as the N-donor ligand because it is a very efficient ligand and is very suitable to coordinate to the central atom. Besides,its triazine ring is capable of coordinating to metal atom, and its amino group can form hydrogen bonds with other acceptors. [Al(CH_3_)_2_]_5_[C_11_H_15_N_8_][Al(CH_3_)_3_] is the only crystallographic complex of ADMT reported so far (Self *et al.*,1991). As a part of the extension of our study on the emission of silver (I) complexes of heterocyclic-N ligands, we synthesized the first silver (I) complex of ADMT, [Ag(ADMT)_3_]_2_(OTf)_2_.(ADMT)_2_.

The title complex consists of two [Ag(ADMT)_3_] cations,two free OTf^-^ anions and two free ADMT ligands. In the complex, the angles N—Ag—N are in the range of 118.6 (2)–121.8 (3)°, which confirms the approximate plane coordination environment around the silver atom. The Ag(I) atom is coordinated by three N atoms from ADMT. The three Ag—N distances are in the range of 2.241 (6)–2.273 (6) Å, which agree with those in [Ag(µ-admtrz)(C_6_H_5_COO)]_2_.2H_2_O (2.2460 (19)–2.251 (2) Å)(Wang & Cheng, 2007) and are slightly longer than those in [Ag_2_(admtrz)_2_(CF_3_CO_2_)_2_] (2.205 (2)–2.250 (2) Å)(Liu *et al.*, 2002).

We also tried to synthesize more silver(I) complexes of ADMT, but failed. When starting material AgOTf was replaced by AgBr, only crystalized ADMT ligand was obtained, and when the title compound was further reacting with PPh_3_, only the Ag:PPh3(1:4) adduct was obtained (Jiang *et al.*, 2011). The failure of the above reactions maybe is because the coordination ability of ADMT to silver(I) is weaker than PPh_3_ ligand and the bromide ion.

### Experimental

A mixture of AgOTf (silver trifluoromethanesulfonate) and ADMT (ADMT = 3-Amino-5,6-dimethyl-1,2,4-triazine) in molar ratio of 2:3 in the mixed solution of CH_3_CN (5 ml)/ CH_2_Cl_2_(5 ml) was stirred for 6 h at room temperature,then filtered. Subsequent slow evaporation of the filtrate resulted in the formation of yellow crystals of the adduct of AgOTf:ADMT(1:4). Yellow prisms were selected directly from the sample as prepared.

### Refinement

All hydrogen atoms were located in the calculated sites and included in the final refinement in the riding model approximation with displacement parameters derived from the parent atoms to which they were bonded.

### Crystal data

**Table:**

[Ag(C_5_H_8_N_4_)_3_](CF_3_O_3_S)·C_5_H_8_N_4_	*Z* = 4
*M**_r_* = 753.56	*F*(000) = 1536
Triclinic, *P*1	*D*_x_ = 1.578 Mg m^−^^3^
Hall symbol: -P 1	Mo *K*α radiation, λ = 0.71073 Å
*a* = 13.9357 (14) Å	Cell parameters from 2772 reflections
*b* = 15.1693 (15) Å	θ = 2.7–20.9°
*c* = 16.2257 (17) Å	µ = 0.77 mm^−^^1^
α = 76.735 (1)°	*T* = 298 K
β = 72.565 (1)°	Prism, yellow
γ = 81.201 (2)°	0.45 × 0.27 × 0.23 mm
*V* = 3172.0 (6) Å^3^	

### Data collection

**Table:**

Bruker SMART CCD diffractometer	11051 independent reflections
Radiation source: fine-focus sealed tube	4939 reflections with *I* > 2σ(*I*)
graphite	*R*_int_ = 0.051
φ and ω scans	θ_max_ = 25.0°, θ_min_ = 2.6°
Absorption correction: multi-scan (*SADABS*; Bruker, 2007)	*h* = −16→16
*T*_min_ = 0.723, *T*_max_ = 0.843	*k* = −18→17
16692 measured reflections	*l* = −19→19

### Refinement

**Table:**

Refinement on *F*^2^	Primary atom site location: structure-invariant direct methods
Least-squares matrix: full	Secondary atom site location: difference Fourier map
*R*[*F*^2^ > 2σ(*F*^2^)] = 0.069	Hydrogen site location: inferred from neighbouring sites
*wR*(*F*^2^) = 0.192	H-atom parameters constrained
*S* = 1.05	*w* = 1/[σ^2^(*F*_o_^2^) + (0.0693*P*)^2^] where *P* = (*F*_o_^2^ + 2*F*_c_^2^)/3
11051 reflections	(Δ/σ)_max_ = 0.001
827 parameters	Δρ_max_ = 0.91 e Å^−^^3^
0 restraints	Δρ_min_ = −0.62 e Å^−^^3^

### Special details

**Table:**

Geometry. All e.s.d.'s (except the e.s.d. in the dihedral angle between two l.s. planes) are estimated using the full covariance matrix. The cell e.s.d.'s are taken into account individually in the estimation of e.s.d.'s in distances, angles and torsion angles; correlations between e.s.d.'s in cell parameters are only used when they are defined by crystal symmetry. An approximate (isotropic) treatment of cell e.s.d.'s is used for estimating e.s.d.'s involving l.s. planes.
Refinement. Refinement of *F*^2^ against ALL reflections. The weighted *R*-factor *wR* and goodness of fit *S* are based on *F*^2^, conventional *R*-factors *R* are based on *F*, with *F* set to zero for negative *F*^2^. The threshold expression of *F*^2^ > σ(*F*^2^) is used only for calculating *R*-factors(gt) *etc*. and is not relevant to the choice of reflections for refinement. *R*-factors based on *F*^2^ are statistically about twice as large as those based on *F*, and *R*- factors based on ALL data will be even larger.

### Fractional atomic coordinates and isotropic or equivalent isotropic displacement parameters (Å^2^)

**Table:**

	*x*	*y*	*z*	*U*_iso_*/*U*_eq_	
Ag1	0.85834 (6)	0.57530 (4)	0.44760 (4)	0.0605 (3)	
Ag2	0.62635 (6)	0.57511 (4)	0.39966 (4)	0.0590 (3)	
F1	0.3350 (8)	−0.0570 (6)	0.2406 (5)	0.163 (4)	
F2	0.2818 (8)	0.0807 (7)	0.2091 (6)	0.190 (5)	
F3	0.4294 (9)	0.0428 (6)	0.1604 (6)	0.195 (5)	
F4	0.7901 (9)	0.1723 (6)	0.0993 (7)	0.198 (5)	
F5	0.8296 (10)	0.2132 (7)	−0.0413 (7)	0.211 (5)	
F6	0.9350 (11)	0.1606 (7)	0.0250 (7)	0.236 (7)	
N1	0.9816 (5)	0.4714 (5)	0.3048 (5)	0.0559 (19)	
N2	0.9583 (5)	0.4562 (5)	0.3925 (4)	0.0520 (19)	
N3	1.0454 (5)	0.3090 (5)	0.3950 (5)	0.058 (2)	
N4	0.9681 (5)	0.3603 (4)	0.5234 (4)	0.059 (2)	
H4A	0.9343	0.4018	0.5516	0.071*	
H4B	0.9878	0.3089	0.5517	0.071*	
N5	0.8133 (5)	0.4731 (4)	0.6360 (4)	0.0486 (18)	
N6	0.7866 (5)	0.5570 (4)	0.5936 (4)	0.0492 (18)	
N7	0.7011 (5)	0.5999 (5)	0.7311 (4)	0.0513 (19)	
N8	0.7078 (5)	0.7007 (5)	0.6006 (4)	0.0558 (19)	
H8A	0.7279	0.7150	0.5441	0.067*	
H8B	0.6725	0.7403	0.6306	0.067*	
N9	0.7701 (6)	0.7679 (5)	0.3997 (5)	0.063 (2)	
N10	0.8303 (5)	0.7047 (5)	0.3552 (4)	0.0539 (19)	
N11	0.8486 (6)	0.8106 (6)	0.2206 (5)	0.066 (2)	
N12	0.9279 (6)	0.6665 (5)	0.2261 (5)	0.070 (2)	
H12A	0.9532	0.6794	0.1699	0.084*	
H12B	0.9414	0.6134	0.2551	0.084*	
N13	0.7109 (6)	0.6255 (5)	0.2021 (4)	0.061 (2)	
N14	0.6504 (6)	0.6667 (5)	0.2660 (4)	0.0538 (19)	
N15	0.6378 (6)	0.8034 (5)	0.1625 (5)	0.068 (2)	
N16	0.5526 (6)	0.7949 (5)	0.3078 (5)	0.067 (2)	
H16A	0.5299	0.8508	0.2950	0.081*	
H16B	0.5357	0.7654	0.3612	0.081*	
N17	0.5076 (5)	0.7193 (5)	0.5054 (4)	0.0526 (19)	
N18	0.5390 (5)	0.6308 (4)	0.5218 (4)	0.0443 (17)	
N19	0.4663 (5)	0.6253 (5)	0.6749 (4)	0.0463 (17)	
N20	0.5450 (5)	0.4976 (4)	0.6228 (4)	0.0516 (19)	
H20A	0.5766	0.4694	0.5807	0.062*	
H20B	0.5312	0.4684	0.6765	0.062*	
N21	0.6949 (5)	0.3913 (5)	0.4975 (4)	0.0482 (18)	
N22	0.7082 (5)	0.4332 (4)	0.4127 (4)	0.0491 (18)	
N23	0.8044 (5)	0.2997 (5)	0.3675 (5)	0.0542 (19)	
N24	0.7741 (5)	0.4263 (5)	0.2654 (4)	0.061 (2)	
H24A	0.8071	0.3974	0.2242	0.074*	
H24B	0.7483	0.4812	0.2523	0.074*	
N25	0.4412 (6)	0.6771 (6)	0.1733 (5)	0.070 (2)	
N26	0.4880 (6)	0.5936 (6)	0.1872 (4)	0.064 (2)	
N27	0.5599 (6)	0.6042 (6)	0.0330 (5)	0.074 (2)	
N28	0.5937 (6)	0.4778 (6)	0.1324 (5)	0.085 (3)	
H28A	0.5871	0.4474	0.1852	0.102*	
H28B	0.6315	0.4549	0.0884	0.102*	
N29	1.0542 (8)	0.8444 (7)	0.3046 (7)	0.095 (3)	
N30	1.0023 (7)	0.8291 (6)	0.3872 (7)	0.086 (3)	
N31	0.9240 (7)	0.9816 (6)	0.3714 (7)	0.092 (3)	
N32	0.8858 (7)	0.8821 (6)	0.5052 (6)	0.099 (3)	
H32A	0.8434	0.9241	0.5267	0.119*	
H32B	0.8937	0.8298	0.5381	0.119*	
O1	0.3892 (7)	0.1421 (5)	0.3072 (5)	0.115 (3)	
O2	0.2964 (7)	0.0192 (6)	0.3926 (5)	0.139 (3)	
O3	0.4723 (7)	−0.0104 (5)	0.3231 (6)	0.129 (3)	
O4	0.8797 (9)	0.3176 (7)	0.1157 (6)	0.169 (4)	
O5	0.7677 (10)	0.3640 (8)	0.0392 (7)	0.214 (6)	
O6	0.9379 (8)	0.3509 (6)	−0.0429 (5)	0.149 (4)	
S1	0.3813 (2)	0.04741 (18)	0.32365 (17)	0.0701 (8)	
S2	0.8694 (3)	0.3218 (2)	0.03687 (17)	0.0847 (10)	
C1	0.9898 (7)	0.3756 (6)	0.4356 (6)	0.052 (2)	
C2	1.0662 (7)	0.3246 (6)	0.3096 (7)	0.065 (3)	
C3	1.0311 (7)	0.4084 (7)	0.2618 (6)	0.058 (3)	
C4	1.1296 (8)	0.2516 (7)	0.2610 (7)	0.103 (4)	
H4C	1.1978	0.2677	0.2361	0.154*	
H4D	1.1021	0.2468	0.2147	0.154*	
H4E	1.1289	0.1944	0.3013	0.154*	
C5	1.0511 (8)	0.4285 (7)	0.1647 (6)	0.084 (3)	
H5A	1.0256	0.4900	0.1458	0.127*	
H5B	1.0181	0.3875	0.1474	0.127*	
H5C	1.1226	0.4211	0.1379	0.127*	
C6	0.7315 (6)	0.6171 (6)	0.6418 (5)	0.048 (2)	
C7	0.7255 (7)	0.5173 (6)	0.7707 (5)	0.052 (2)	
C8	0.7839 (6)	0.4525 (6)	0.7222 (5)	0.049 (2)	
C9	0.6924 (8)	0.4960 (6)	0.8691 (5)	0.077 (3)	
H9A	0.6467	0.5452	0.8904	0.115*	
H9B	0.7504	0.4883	0.8913	0.115*	
H9C	0.6591	0.4410	0.8888	0.115*	
C10	0.8154 (7)	0.3597 (6)	0.7640 (5)	0.070 (3)	
H10A	0.8526	0.3260	0.7192	0.105*	
H10B	0.7568	0.3296	0.7997	0.105*	
H10C	0.8575	0.3633	0.8002	0.105*	
C11	0.8691 (7)	0.7275 (7)	0.2671 (6)	0.059 (3)	
C12	0.7896 (8)	0.8702 (7)	0.2633 (7)	0.067 (3)	
C13	0.7515 (8)	0.8503 (7)	0.3542 (7)	0.065 (3)	
C14	0.7622 (9)	0.9608 (6)	0.2120 (7)	0.098 (4)	
H14A	0.8102	0.9711	0.1550	0.147*	
H14B	0.7627	1.0075	0.2428	0.147*	
H14C	0.6959	0.9618	0.2051	0.147*	
C15	0.6843 (8)	0.9157 (6)	0.4067 (7)	0.084 (3)	
H15A	0.6615	0.8858	0.4671	0.126*	
H15B	0.6272	0.9378	0.3839	0.126*	
H15C	0.7210	0.9659	0.4029	0.126*	
C16	0.6146 (7)	0.7535 (7)	0.2442 (6)	0.061 (3)	
C17	0.7000 (8)	0.7623 (8)	0.1020 (6)	0.074 (3)	
C18	0.7366 (7)	0.6708 (7)	0.1215 (6)	0.064 (3)	
C19	0.7335 (10)	0.8184 (8)	0.0091 (6)	0.116 (5)	
H19A	0.7058	0.8804	0.0090	0.175*	
H19B	0.7099	0.7942	−0.0302	0.175*	
H19C	0.8059	0.8160	−0.0099	0.175*	
C20	0.8052 (8)	0.6208 (7)	0.0535 (6)	0.096 (4)	
H20C	0.8217	0.5595	0.0811	0.144*	
H20D	0.8659	0.6508	0.0260	0.144*	
H20E	0.7718	0.6198	0.0098	0.144*	
C21	0.5172 (6)	0.5868 (6)	0.6048 (5)	0.046 (2)	
C22	0.4371 (6)	0.7125 (6)	0.6571 (5)	0.048 (2)	
C23	0.4594 (6)	0.7599 (6)	0.5694 (5)	0.050 (2)	
C24	0.3812 (7)	0.7587 (6)	0.7333 (5)	0.071 (3)	
H24C	0.3665	0.7139	0.7867	0.107*	
H24D	0.3193	0.7901	0.7232	0.107*	
H24E	0.4223	0.8014	0.7383	0.107*	
C25	0.4255 (7)	0.8606 (5)	0.5461 (6)	0.070 (3)	
H25A	0.4486	0.8812	0.4836	0.105*	
H25B	0.4533	0.8945	0.5752	0.105*	
H25C	0.3531	0.8694	0.5649	0.105*	
C26	0.7627 (6)	0.3861 (6)	0.3496 (5)	0.049 (2)	
C27	0.7902 (7)	0.2612 (6)	0.4510 (6)	0.053 (2)	
C28	0.7321 (7)	0.3089 (6)	0.5175 (6)	0.052 (2)	
C29	0.8393 (7)	0.1653 (6)	0.4724 (6)	0.076 (3)	
H29A	0.8638	0.1403	0.4199	0.114*	
H29B	0.7905	0.1280	0.5154	0.114*	
H29C	0.8947	0.1670	0.4956	0.114*	
C30	0.7119 (7)	0.2661 (6)	0.6133 (5)	0.072 (3)	
H30A	0.6754	0.3102	0.6481	0.108*	
H30B	0.7748	0.2453	0.6273	0.108*	
H30C	0.6725	0.2156	0.6259	0.108*	
C31	0.5450 (8)	0.5607 (7)	0.1187 (6)	0.068 (3)	
C32	0.5135 (8)	0.6842 (7)	0.0203 (6)	0.075 (3)	
C33	0.4539 (8)	0.7238 (7)	0.0937 (7)	0.073 (3)	
C34	0.5243 (10)	0.7321 (8)	−0.0731 (7)	0.119 (5)	
H34A	0.5519	0.6895	−0.1116	0.179*	
H34B	0.5686	0.7793	−0.0877	0.179*	
H34C	0.4592	0.7583	−0.0799	0.179*	
C35	0.4012 (9)	0.8178 (8)	0.0817 (7)	0.112 (4)	
H35A	0.3554	0.8211	0.0470	0.168*	
H35B	0.4503	0.8610	0.0522	0.168*	
H35C	0.3643	0.8316	0.1382	0.168*	
C36	0.9397 (10)	0.8981 (8)	0.4200 (8)	0.085 (3)	
C37	0.9790 (10)	0.9965 (8)	0.2892 (9)	0.096 (4)	
C38	1.0467 (10)	0.9251 (10)	0.2541 (9)	0.097 (4)	
C39	0.9637 (11)	1.0926 (8)	0.2341 (9)	0.141 (6)	
H39A	0.8928	1.1098	0.2419	0.212*	
H39B	0.9964	1.0923	0.1730	0.212*	
H39C	0.9924	1.1353	0.2531	0.212*	
C40	1.1061 (11)	0.9389 (9)	0.1612 (9)	0.152 (6)	
H40A	1.1435	0.8827	0.1485	0.228*	
H40B	1.1520	0.9841	0.1502	0.228*	
H40C	1.0614	0.9589	0.1242	0.228*	
C41	0.3497 (14)	0.0270 (10)	0.2315 (10)	0.119 (5)	
C42	0.8448 (17)	0.2119 (12)	0.0317 (11)	0.141 (6)	

### Atomic displacement parameters (Å^2^)

**Table:**

	*U*^11^	*U*^22^	*U*^33^	*U*^12^	*U*^13^	*U*^23^
Ag1	0.0740 (6)	0.0538 (5)	0.0445 (4)	0.0110 (4)	−0.0075 (4)	−0.0140 (3)
Ag2	0.0749 (6)	0.0506 (4)	0.0435 (4)	0.0070 (4)	−0.0073 (4)	−0.0135 (3)
F1	0.252 (11)	0.104 (6)	0.166 (8)	−0.009 (7)	−0.089 (7)	−0.056 (5)
F2	0.249 (12)	0.172 (9)	0.200 (9)	0.077 (8)	−0.161 (10)	−0.068 (7)
F3	0.287 (14)	0.167 (8)	0.085 (6)	0.017 (9)	−0.001 (7)	−0.027 (5)
F4	0.245 (13)	0.124 (7)	0.176 (9)	−0.063 (8)	0.002 (9)	0.019 (6)
F5	0.319 (16)	0.196 (10)	0.153 (9)	−0.064 (10)	−0.087 (10)	−0.047 (7)
F6	0.284 (15)	0.157 (9)	0.183 (10)	0.088 (10)	−0.005 (10)	−0.017 (7)
N1	0.052 (5)	0.061 (5)	0.054 (5)	−0.003 (4)	−0.008 (4)	−0.020 (4)
N2	0.048 (5)	0.060 (5)	0.052 (5)	0.002 (4)	−0.010 (4)	−0.027 (4)
N3	0.052 (5)	0.059 (5)	0.068 (5)	0.007 (4)	−0.015 (4)	−0.033 (4)
N4	0.067 (6)	0.047 (4)	0.054 (5)	0.018 (4)	−0.014 (4)	−0.014 (3)
N5	0.045 (5)	0.049 (4)	0.046 (4)	−0.001 (4)	−0.006 (3)	−0.010 (3)
N6	0.058 (5)	0.044 (4)	0.040 (4)	0.002 (4)	−0.003 (4)	−0.017 (3)
N7	0.049 (5)	0.060 (5)	0.042 (4)	−0.005 (4)	−0.003 (4)	−0.018 (4)
N8	0.057 (5)	0.056 (5)	0.049 (4)	0.010 (4)	−0.007 (4)	−0.018 (4)
N9	0.066 (6)	0.056 (5)	0.066 (5)	0.005 (4)	−0.019 (4)	−0.019 (4)
N10	0.055 (5)	0.051 (5)	0.055 (5)	−0.002 (4)	−0.019 (4)	−0.006 (4)
N11	0.063 (6)	0.069 (6)	0.063 (5)	−0.008 (5)	−0.024 (4)	0.004 (5)
N12	0.067 (6)	0.077 (6)	0.050 (5)	−0.001 (5)	0.002 (4)	−0.010 (4)
N13	0.067 (6)	0.074 (5)	0.041 (4)	−0.002 (4)	−0.012 (4)	−0.017 (4)
N14	0.060 (5)	0.060 (5)	0.044 (4)	−0.006 (4)	−0.017 (4)	−0.013 (4)
N15	0.082 (7)	0.071 (5)	0.048 (5)	−0.009 (5)	−0.020 (5)	0.002 (4)
N16	0.083 (6)	0.057 (5)	0.056 (5)	0.010 (5)	−0.021 (4)	−0.009 (4)
N17	0.056 (5)	0.048 (5)	0.052 (4)	0.006 (4)	−0.013 (4)	−0.016 (4)
N18	0.046 (5)	0.049 (4)	0.036 (4)	0.006 (4)	−0.006 (3)	−0.018 (3)
N19	0.046 (5)	0.051 (4)	0.042 (4)	0.000 (4)	−0.009 (3)	−0.017 (3)
N20	0.056 (5)	0.050 (5)	0.039 (4)	0.003 (4)	−0.001 (3)	−0.012 (3)
N21	0.045 (5)	0.051 (5)	0.048 (4)	0.010 (4)	−0.016 (4)	−0.016 (4)
N22	0.046 (5)	0.048 (4)	0.053 (4)	0.003 (4)	−0.010 (4)	−0.018 (4)
N23	0.050 (5)	0.052 (5)	0.065 (5)	0.006 (4)	−0.016 (4)	−0.027 (4)
N24	0.066 (6)	0.068 (5)	0.046 (4)	0.009 (4)	−0.007 (4)	−0.023 (4)
N25	0.077 (6)	0.077 (6)	0.058 (5)	0.000 (5)	−0.012 (5)	−0.030 (5)
N26	0.068 (6)	0.075 (6)	0.049 (5)	0.004 (5)	−0.014 (4)	−0.023 (4)
N27	0.084 (7)	0.076 (6)	0.048 (5)	0.009 (5)	−0.008 (4)	−0.011 (4)
N28	0.113 (8)	0.075 (6)	0.053 (5)	0.023 (6)	−0.011 (5)	−0.019 (4)
N29	0.089 (8)	0.090 (8)	0.101 (8)	0.015 (6)	−0.028 (7)	−0.025 (6)
N30	0.083 (7)	0.071 (6)	0.098 (7)	0.014 (6)	−0.027 (6)	−0.015 (6)
N31	0.102 (8)	0.071 (6)	0.098 (7)	0.007 (6)	−0.033 (7)	−0.010 (6)
N32	0.098 (8)	0.084 (7)	0.099 (7)	0.028 (6)	−0.021 (6)	−0.017 (6)
O1	0.150 (8)	0.069 (5)	0.138 (7)	0.010 (5)	−0.045 (6)	−0.048 (5)
O2	0.143 (8)	0.122 (7)	0.111 (7)	−0.015 (6)	0.033 (6)	−0.033 (5)
O3	0.135 (8)	0.108 (6)	0.152 (8)	0.066 (6)	−0.067 (6)	−0.053 (5)
O4	0.227 (12)	0.205 (11)	0.095 (7)	−0.073 (9)	−0.031 (7)	−0.049 (7)
O5	0.198 (13)	0.191 (11)	0.171 (10)	0.092 (10)	−0.010 (9)	0.000 (8)
O6	0.182 (10)	0.146 (8)	0.084 (6)	−0.048 (7)	0.026 (6)	−0.017 (5)
S1	0.087 (2)	0.0575 (17)	0.0635 (16)	0.0187 (16)	−0.0222 (15)	−0.0228 (13)
S2	0.122 (3)	0.0719 (19)	0.0451 (15)	−0.0117 (19)	0.0017 (17)	−0.0132 (13)
C1	0.048 (6)	0.055 (6)	0.058 (6)	0.001 (5)	−0.015 (5)	−0.021 (5)
C2	0.056 (7)	0.061 (7)	0.082 (7)	0.005 (5)	−0.010 (6)	−0.042 (6)
C3	0.053 (7)	0.073 (7)	0.057 (6)	−0.006 (5)	−0.011 (5)	−0.036 (5)
C4	0.109 (10)	0.091 (8)	0.107 (9)	0.016 (7)	−0.010 (7)	−0.062 (7)
C5	0.089 (9)	0.103 (8)	0.058 (6)	−0.008 (7)	0.000 (6)	−0.036 (6)
C6	0.044 (6)	0.054 (6)	0.047 (5)	0.000 (5)	−0.010 (4)	−0.017 (4)
C7	0.056 (6)	0.056 (6)	0.043 (5)	−0.003 (5)	−0.009 (4)	−0.013 (4)
C8	0.049 (6)	0.055 (6)	0.040 (5)	0.005 (5)	−0.011 (4)	−0.014 (4)
C9	0.090 (8)	0.088 (7)	0.049 (6)	−0.009 (6)	−0.007 (5)	−0.021 (5)
C10	0.067 (7)	0.075 (7)	0.054 (6)	0.007 (6)	−0.006 (5)	−0.004 (5)
C11	0.063 (7)	0.063 (7)	0.054 (6)	−0.005 (6)	−0.024 (5)	−0.011 (5)
C12	0.070 (8)	0.057 (7)	0.075 (7)	−0.013 (6)	−0.031 (6)	0.007 (6)
C13	0.068 (8)	0.057 (6)	0.075 (7)	0.006 (6)	−0.033 (6)	−0.011 (5)
C14	0.112 (10)	0.067 (7)	0.107 (9)	−0.007 (7)	−0.043 (8)	0.017 (6)
C15	0.085 (8)	0.060 (7)	0.105 (8)	0.030 (6)	−0.036 (7)	−0.023 (6)
C16	0.067 (7)	0.071 (7)	0.047 (6)	−0.003 (6)	−0.024 (5)	−0.004 (5)
C17	0.079 (8)	0.091 (8)	0.050 (6)	−0.013 (7)	−0.020 (6)	−0.002 (6)
C18	0.065 (7)	0.077 (7)	0.045 (6)	−0.002 (6)	−0.009 (5)	−0.016 (5)
C19	0.139 (12)	0.121 (10)	0.070 (8)	−0.018 (9)	−0.022 (8)	0.016 (7)
C20	0.103 (10)	0.117 (9)	0.057 (6)	−0.010 (8)	−0.002 (6)	−0.020 (6)
C21	0.045 (6)	0.054 (6)	0.039 (5)	−0.005 (5)	−0.007 (4)	−0.019 (4)
C22	0.043 (6)	0.057 (6)	0.049 (5)	0.004 (5)	−0.010 (4)	−0.028 (4)
C23	0.044 (6)	0.054 (6)	0.052 (5)	0.008 (5)	−0.013 (4)	−0.019 (5)
C24	0.077 (8)	0.070 (6)	0.059 (6)	0.011 (6)	−0.006 (5)	−0.025 (5)
C25	0.079 (8)	0.056 (6)	0.064 (6)	0.017 (6)	−0.012 (5)	−0.016 (5)
C26	0.044 (6)	0.052 (6)	0.053 (6)	0.000 (5)	−0.009 (4)	−0.024 (5)
C27	0.056 (6)	0.045 (5)	0.063 (6)	0.001 (5)	−0.021 (5)	−0.018 (5)
C28	0.048 (6)	0.051 (6)	0.060 (6)	0.003 (5)	−0.019 (5)	−0.019 (5)
C29	0.079 (8)	0.059 (6)	0.089 (7)	0.007 (6)	−0.019 (6)	−0.025 (5)
C30	0.074 (8)	0.068 (6)	0.062 (6)	0.015 (6)	−0.014 (5)	−0.011 (5)
C31	0.084 (8)	0.071 (7)	0.044 (6)	0.001 (6)	−0.011 (5)	−0.019 (5)
C32	0.088 (9)	0.074 (7)	0.057 (6)	0.001 (7)	−0.015 (6)	−0.015 (6)
C33	0.080 (8)	0.079 (8)	0.062 (7)	0.002 (6)	−0.020 (6)	−0.026 (6)
C34	0.143 (12)	0.108 (10)	0.078 (8)	0.024 (9)	−0.016 (8)	−0.005 (7)
C35	0.127 (12)	0.095 (9)	0.108 (9)	0.030 (8)	−0.026 (8)	−0.041 (7)
C36	0.090 (10)	0.070 (8)	0.097 (9)	0.003 (7)	−0.037 (8)	−0.015 (7)
C37	0.099 (10)	0.086 (9)	0.095 (9)	−0.001 (8)	−0.027 (8)	−0.004 (8)
C38	0.097 (11)	0.089 (10)	0.100 (10)	0.000 (8)	−0.022 (8)	−0.017 (8)
C39	0.163 (15)	0.092 (10)	0.153 (13)	−0.020 (10)	−0.052 (11)	0.024 (9)
C40	0.160 (15)	0.137 (13)	0.125 (12)	0.007 (11)	−0.007 (11)	−0.013 (10)
C41	0.159 (16)	0.081 (10)	0.124 (12)	0.036 (11)	−0.060 (12)	−0.036 (9)
C42	0.20 (2)	0.149 (16)	0.074 (10)	−0.023 (15)	−0.034 (12)	−0.021 (10)

### Geometric parameters (Å, °)

**Table:**

Ag1—N10	2.241 (6)	O3—S1	1.427 (8)
Ag1—N6	2.248 (6)	O4—S2	1.316 (9)
Ag1—N2	2.287 (7)	O5—S2	1.457 (12)
Ag2—N14	2.251 (7)	O6—S2	1.380 (8)
Ag2—N18	2.264 (6)	S1—C41	1.781 (14)
Ag2—N22	2.273 (6)	S2—C42	1.777 (18)
F1—C41	1.290 (14)	C2—C3	1.430 (12)
F2—C41	1.234 (14)	C2—C4	1.520 (12)
F3—C41	1.345 (17)	C3—C5	1.482 (11)
F4—C42	1.217 (16)	C4—H4C	0.9600
F5—C42	1.260 (15)	C4—H4D	0.9600
F6—C42	1.36 (2)	C4—H4E	0.9600
N1—C3	1.301 (10)	C5—H5A	0.9600
N1—N2	1.334 (8)	C5—H5B	0.9600
N2—C1	1.342 (10)	C5—H5C	0.9600
N3—C2	1.300 (11)	C7—C8	1.405 (11)
N3—C1	1.348 (10)	C7—C9	1.494 (11)
N4—C1	1.336 (9)	C8—C10	1.479 (11)
N4—H4A	0.8600	C9—H9A	0.9600
N4—H4B	0.8600	C9—H9B	0.9600
N5—C8	1.311 (9)	C9—H9C	0.9600
N5—N6	1.355 (8)	C10—H10A	0.9600
N6—C6	1.336 (9)	C10—H10B	0.9600
N7—C7	1.315 (10)	C10—H10C	0.9600
N7—C6	1.357 (9)	C12—C13	1.388 (12)
N8—C6	1.333 (9)	C12—C14	1.493 (12)
N8—H8A	0.8600	C13—C15	1.488 (12)
N8—H8B	0.8600	C14—H14A	0.9600
N9—C13	1.329 (10)	C14—H14B	0.9600
N9—N10	1.362 (9)	C14—H14C	0.9600
N10—C11	1.350 (10)	C15—H15A	0.9600
N11—C12	1.302 (12)	C15—H15B	0.9600
N11—C11	1.349 (11)	C15—H15C	0.9600
N12—C11	1.301 (10)	C17—C18	1.402 (13)
N12—H12A	0.8600	C17—C19	1.524 (12)
N12—H12B	0.8600	C18—C20	1.496 (12)
N13—C18	1.301 (10)	C19—H19A	0.9600
N13—N14	1.338 (9)	C19—H19B	0.9600
N14—C16	1.341 (10)	C19—H19C	0.9600
N15—C17	1.309 (12)	C20—H20C	0.9600
N15—C16	1.338 (10)	C20—H20D	0.9600
N16—C16	1.342 (11)	C20—H20E	0.9600
N16—H16A	0.8600	C22—C23	1.405 (11)
N16—H16B	0.8600	C22—C24	1.509 (10)
N17—C23	1.288 (9)	C23—C25	1.522 (11)
N17—N18	1.339 (8)	C24—H24C	0.9600
N18—C21	1.325 (9)	C24—H24D	0.9600
N19—C22	1.315 (9)	C24—H24E	0.9600
N19—C21	1.354 (9)	C25—H25A	0.9600
N20—C21	1.340 (9)	C25—H25B	0.9600
N20—H20A	0.8600	C25—H25C	0.9600
N20—H20B	0.8600	C27—C28	1.414 (11)
N21—C28	1.287 (9)	C27—C29	1.521 (11)
N21—N22	1.346 (8)	C28—C30	1.499 (11)
N22—C26	1.356 (9)	C29—H29A	0.9600
N23—C27	1.314 (10)	C29—H29B	0.9600
N23—C26	1.354 (10)	C29—H29C	0.9600
N24—C26	1.335 (9)	C30—H30A	0.9600
N24—H24A	0.8600	C30—H30B	0.9600
N24—H24B	0.8600	C30—H30C	0.9600
N25—C33	1.298 (11)	C32—C33	1.433 (13)
N25—N26	1.335 (9)	C32—C34	1.496 (13)
N26—C31	1.311 (10)	C33—C35	1.500 (13)
N27—C32	1.287 (11)	C34—H34A	0.9600
N27—C31	1.364 (10)	C34—H34B	0.9600
N28—C31	1.338 (11)	C34—H34C	0.9600
N28—H28A	0.8600	C35—H35A	0.9600
N28—H28B	0.8600	C35—H35B	0.9600
N29—N30	1.304 (11)	C35—H35C	0.9600
N29—C38	1.316 (13)	C37—C38	1.427 (15)
N30—C36	1.355 (13)	C37—C39	1.546 (14)
N31—C37	1.311 (13)	C38—C40	1.470 (15)
N31—C36	1.354 (12)	C39—H39A	0.9600
N32—C36	1.347 (12)	C39—H39B	0.9600
N32—H32A	0.8600	C39—H39C	0.9600
N32—H32B	0.8600	C40—H40A	0.9600
O1—S1	1.415 (7)	C40—H40B	0.9600
O2—S1	1.405 (8)	C40—H40C	0.9600
			
N10—Ag1—N6	121.8 (3)	C12—C14—H14A	109.5
N10—Ag1—N2	119.6 (3)	C12—C14—H14B	109.5
N6—Ag1—N2	118.6 (2)	H14A—C14—H14B	109.5
N14—Ag2—N18	119.9 (3)	C12—C14—H14C	109.5
N14—Ag2—N22	119.5 (3)	H14A—C14—H14C	109.5
N18—Ag2—N22	120.0 (2)	H14B—C14—H14C	109.5
C3—N1—N2	120.7 (8)	C13—C15—H15A	109.5
N1—N2—C1	118.8 (7)	C13—C15—H15B	109.5
N1—N2—Ag1	111.7 (5)	H15A—C15—H15B	109.5
C1—N2—Ag1	129.4 (6)	C13—C15—H15C	109.5
C2—N3—C1	116.8 (8)	H15A—C15—H15C	109.5
C1—N4—H4A	120.0	H15B—C15—H15C	109.5
C1—N4—H4B	120.0	N15—C16—N14	124.5 (9)
H4A—N4—H4B	120.0	N15—C16—N16	116.7 (9)
C8—N5—N6	119.7 (7)	N14—C16—N16	118.8 (8)
C6—N6—N5	118.5 (7)	N15—C17—C18	121.7 (9)
C6—N6—Ag1	129.8 (6)	N15—C17—C19	117.4 (10)
N5—N6—Ag1	111.4 (5)	C18—C17—C19	120.9 (10)
C7—N7—C6	116.4 (7)	N13—C18—C17	119.8 (9)
C6—N8—H8A	120.0	N13—C18—C20	117.0 (9)
C6—N8—H8B	120.0	C17—C18—C20	123.2 (9)
H8A—N8—H8B	120.0	C17—C19—H19A	109.5
C13—N9—N10	118.3 (8)	C17—C19—H19B	109.5
C11—N10—N9	119.4 (7)	H19A—C19—H19B	109.5
C11—N10—Ag1	129.3 (6)	C17—C19—H19C	109.5
N9—N10—Ag1	111.2 (5)	H19A—C19—H19C	109.5
C12—N11—C11	118.0 (9)	H19B—C19—H19C	109.5
C11—N12—H12A	120.0	C18—C20—H20C	109.5
C11—N12—H12B	120.0	C18—C20—H20D	109.5
H12A—N12—H12B	120.0	H20C—C20—H20D	109.5
C18—N13—N14	119.8 (8)	C18—C20—H20E	109.5
N13—N14—C16	118.5 (7)	H20C—C20—H20E	109.5
N13—N14—Ag2	112.2 (5)	H20D—C20—H20E	109.5
C16—N14—Ag2	129.3 (6)	N18—C21—N20	119.3 (7)
C17—N15—C16	115.6 (9)	N18—C21—N19	124.7 (8)
C16—N16—H16A	120.0	N20—C21—N19	116.0 (7)
C16—N16—H16B	120.0	N19—C22—C23	119.9 (7)
H16A—N16—H16B	120.0	N19—C22—C24	117.9 (7)
C23—N17—N18	120.2 (7)	C23—C22—C24	122.1 (8)
C21—N18—N17	118.0 (6)	N17—C23—C22	121.1 (8)
C21—N18—Ag2	128.0 (5)	N17—C23—C25	117.5 (7)
N17—N18—Ag2	113.9 (5)	C22—C23—C25	121.4 (7)
C22—N19—C21	116.0 (7)	C22—C24—H24C	109.5
C21—N20—H20A	120.0	C22—C24—H24D	109.5
C21—N20—H20B	120.0	H24C—C24—H24D	109.5
H20A—N20—H20B	120.0	C22—C24—H24E	109.5
C28—N21—N22	120.7 (7)	H24C—C24—H24E	109.5
N21—N22—C26	118.0 (7)	H24D—C24—H24E	109.5
N21—N22—Ag2	112.0 (5)	C23—C25—H25A	109.5
C26—N22—Ag2	130.0 (6)	C23—C25—H25B	109.5
C27—N23—C26	116.9 (7)	H25A—C25—H25B	109.5
C26—N24—H24A	120.0	C23—C25—H25C	109.5
C26—N24—H24B	120.0	H25A—C25—H25C	109.5
H24A—N24—H24B	120.0	H25B—C25—H25C	109.5
C33—N25—N26	119.9 (8)	N24—C26—N23	118.2 (7)
C31—N26—N25	118.4 (8)	N24—C26—N22	118.3 (8)
C32—N27—C31	115.9 (8)	N23—C26—N22	123.5 (8)
C31—N28—H28A	120.0	N23—C27—C28	120.3 (8)
C31—N28—H28B	120.0	N23—C27—C29	117.7 (8)
H28A—N28—H28B	120.0	C28—C27—C29	122.0 (8)
N30—N29—C38	120.9 (11)	N21—C28—C27	120.7 (8)
N29—N30—C36	118.5 (9)	N21—C28—C30	117.5 (8)
C37—N31—C36	116.4 (11)	C27—C28—C30	121.8 (8)
C36—N32—H32A	120.0	C27—C29—H29A	109.5
C36—N32—H32B	120.0	C27—C29—H29B	109.5
H32A—N32—H32B	120.0	H29A—C29—H29B	109.5
O2—S1—O1	113.3 (5)	C27—C29—H29C	109.5
O2—S1—O3	115.2 (5)	H29A—C29—H29C	109.5
O1—S1—O3	116.6 (6)	H29B—C29—H29C	109.5
O2—S1—C41	101.2 (8)	C28—C30—H30A	109.5
O1—S1—C41	105.7 (6)	C28—C30—H30B	109.5
O3—S1—C41	102.3 (6)	H30A—C30—H30B	109.5
O4—S2—O6	126.8 (7)	C28—C30—H30C	109.5
O4—S2—O5	105.8 (7)	H30A—C30—H30C	109.5
O6—S2—O5	111.8 (6)	H30B—C30—H30C	109.5
O4—S2—C42	107.6 (7)	N26—C31—N28	118.5 (9)
O6—S2—C42	104.8 (7)	N26—C31—N27	125.3 (10)
O5—S2—C42	95.6 (10)	N28—C31—N27	116.1 (9)
N4—C1—N2	119.2 (8)	N27—C32—C33	120.3 (9)
N4—C1—N3	117.2 (8)	N27—C32—C34	117.3 (9)
N2—C1—N3	123.5 (8)	C33—C32—C34	122.5 (10)
N3—C2—C3	121.1 (8)	N25—C33—C32	120.1 (10)
N3—C2—C4	118.6 (9)	N25—C33—C35	117.9 (9)
C3—C2—C4	120.3 (9)	C32—C33—C35	121.9 (10)
N1—C3—C2	118.9 (8)	C32—C34—H34A	109.5
N1—C3—C5	118.0 (9)	C32—C34—H34B	109.5
C2—C3—C5	123.1 (9)	H34A—C34—H34B	109.5
C2—C4—H4C	109.5	C32—C34—H34C	109.5
C2—C4—H4D	109.5	H34A—C34—H34C	109.5
H4C—C4—H4D	109.5	H34B—C34—H34C	109.5
C2—C4—H4E	109.5	C33—C35—H35A	109.5
H4C—C4—H4E	109.5	C33—C35—H35B	109.5
H4D—C4—H4E	109.5	H35A—C35—H35B	109.5
C3—C5—H5A	109.5	C33—C35—H35C	109.5
C3—C5—H5B	109.5	H35A—C35—H35C	109.5
H5A—C5—H5B	109.5	H35B—C35—H35C	109.5
C3—C5—H5C	109.5	N32—C36—N31	117.3 (12)
H5A—C5—H5C	109.5	N32—C36—N30	118.5 (11)
H5B—C5—H5C	109.5	N31—C36—N30	124.1 (12)
N8—C6—N6	118.8 (7)	N31—C37—C38	120.1 (11)
N8—C6—N7	117.1 (8)	N31—C37—C39	116.7 (12)
N6—C6—N7	124.0 (8)	C38—C37—C39	123.2 (13)
N7—C7—C8	121.0 (7)	N29—C38—C37	119.8 (12)
N7—C7—C9	117.7 (8)	N29—C38—C40	119.0 (13)
C8—C7—C9	121.2 (8)	C37—C38—C40	121.2 (12)
N5—C8—C7	120.2 (8)	C37—C39—H39A	109.5
N5—C8—C10	116.9 (7)	C37—C39—H39B	109.5
C7—C8—C10	122.9 (7)	H39A—C39—H39B	109.5
C7—C9—H9A	109.5	C37—C39—H39C	109.5
C7—C9—H9B	109.5	H39A—C39—H39C	109.5
H9A—C9—H9B	109.5	H39B—C39—H39C	109.5
C7—C9—H9C	109.5	C38—C40—H40A	109.5
H9A—C9—H9C	109.5	C38—C40—H40B	109.5
H9B—C9—H9C	109.5	H40A—C40—H40B	109.5
C8—C10—H10A	109.5	C38—C40—H40C	109.5
C8—C10—H10B	109.5	H40A—C40—H40C	109.5
H10A—C10—H10B	109.5	H40B—C40—H40C	109.5
C8—C10—H10C	109.5	F2—C41—F1	113.1 (16)
H10A—C10—H10C	109.5	F2—C41—F3	102.6 (13)
H10B—C10—H10C	109.5	F1—C41—F3	105.4 (13)
N12—C11—N11	119.3 (9)	F2—C41—S1	114.6 (11)
N12—C11—N10	118.3 (9)	F1—C41—S1	111.9 (10)
N11—C11—N10	122.4 (9)	F3—C41—S1	108.2 (13)
N11—C12—C13	121.1 (9)	F4—C42—F5	120 (2)
N11—C12—C14	118.4 (10)	F4—C42—F6	103.2 (16)
C13—C12—C14	120.4 (10)	F5—C42—F6	101.8 (16)
N9—C13—C12	120.8 (9)	F4—C42—S2	114.5 (12)
N9—C13—C15	115.6 (9)	F5—C42—S2	110.1 (13)
C12—C13—C15	123.6 (9)	F6—C42—S2	105.1 (15)
			
C3—N1—N2—C1	2.2 (12)	N13—N14—C16—N15	−2.2 (13)
C3—N1—N2—Ag1	−174.4 (6)	Ag2—N14—C16—N15	176.5 (6)
N10—Ag1—N2—N1	−8.4 (6)	N13—N14—C16—N16	179.0 (8)
N6—Ag1—N2—N1	169.6 (5)	Ag2—N14—C16—N16	−2.3 (13)
N10—Ag1—N2—C1	175.5 (7)	C16—N15—C17—C18	2.3 (15)
N6—Ag1—N2—C1	−6.6 (8)	C16—N15—C17—C19	−176.3 (9)
C8—N5—N6—C6	0.8 (11)	N14—N13—C18—C17	−0.6 (14)
C8—N5—N6—Ag1	175.4 (6)	N14—N13—C18—C20	179.5 (8)
N10—Ag1—N6—C6	−7.1 (8)	N15—C17—C18—N13	−2.0 (16)
N2—Ag1—N6—C6	175.0 (7)	C19—C17—C18—N13	176.6 (10)
N10—Ag1—N6—N5	179.1 (5)	N15—C17—C18—C20	178.0 (10)
N2—Ag1—N6—N5	1.2 (6)	C19—C17—C18—C20	−3.5 (16)
C13—N9—N10—C11	0.2 (12)	N17—N18—C21—N20	178.0 (7)
C13—N9—N10—Ag1	176.0 (6)	Ag2—N18—C21—N20	−2.7 (11)
N6—Ag1—N10—C11	178.0 (7)	N17—N18—C21—N19	−1.0 (12)
N2—Ag1—N10—C11	−4.2 (8)	Ag2—N18—C21—N19	178.4 (6)
N6—Ag1—N10—N9	2.6 (6)	C22—N19—C21—N18	0.4 (12)
N2—Ag1—N10—N9	−179.5 (5)	C22—N19—C21—N20	−178.6 (7)
C18—N13—N14—C16	2.6 (12)	C21—N19—C22—C23	−0.2 (12)
C18—N13—N14—Ag2	−176.4 (7)	C21—N19—C22—C24	−179.3 (7)
N18—Ag2—N14—N13	174.4 (5)	N18—N17—C23—C22	−1.2 (13)
N22—Ag2—N14—N13	2.9 (6)	N18—N17—C23—C25	−179.7 (7)
N18—Ag2—N14—C16	−4.5 (8)	N19—C22—C23—N17	0.6 (13)
N22—Ag2—N14—C16	−176.0 (7)	C24—C22—C23—N17	179.7 (8)
C23—N17—N18—C21	1.3 (11)	N19—C22—C23—C25	179.1 (8)
C23—N17—N18—Ag2	−178.1 (6)	C24—C22—C23—C25	−1.8 (13)
N14—Ag2—N18—C21	179.4 (6)	C27—N23—C26—N24	−178.6 (8)
N22—Ag2—N18—C21	−9.2 (8)	C27—N23—C26—N22	−0.1 (12)
N14—Ag2—N18—N17	−1.2 (6)	N21—N22—C26—N24	178.3 (7)
N22—Ag2—N18—N17	170.2 (5)	Ag2—N22—C26—N24	1.3 (12)
C28—N21—N22—C26	−0.5 (11)	N21—N22—C26—N23	−0.3 (12)
C28—N21—N22—Ag2	177.0 (6)	Ag2—N22—C26—N23	−177.3 (6)
N14—Ag2—N22—N21	172.9 (5)	C26—N23—C27—C28	1.1 (12)
N18—Ag2—N22—N21	1.4 (6)	C26—N23—C27—C29	−178.0 (8)
N14—Ag2—N22—C26	−9.9 (8)	N22—N21—C28—C27	1.7 (12)
N18—Ag2—N22—C26	178.6 (7)	N22—N21—C28—C30	−178.6 (7)
C33—N25—N26—C31	−0.9 (13)	N23—C27—C28—N21	−2.0 (13)
C38—N29—N30—C36	0.5 (17)	C29—C27—C28—N21	177.1 (8)
N1—N2—C1—N4	179.0 (7)	N23—C27—C28—C30	178.2 (8)
Ag1—N2—C1—N4	−5.1 (12)	C29—C27—C28—C30	−2.6 (13)
N1—N2—C1—N3	1.1 (12)	N25—N26—C31—N28	178.6 (8)
Ag1—N2—C1—N3	177.0 (6)	N25—N26—C31—N27	−1.1 (15)
C2—N3—C1—N4	−179.9 (8)	C32—N27—C31—N26	0.2 (16)
C2—N3—C1—N2	−1.9 (13)	C32—N27—C31—N28	−179.5 (9)
C1—N3—C2—C3	−0.2 (13)	C31—N27—C32—C33	2.5 (15)
C1—N3—C2—C4	179.5 (8)	C31—N27—C32—C34	−177.2 (10)
N2—N1—C3—C2	−4.2 (13)	N26—N25—C33—C32	3.5 (15)
N2—N1—C3—C5	177.7 (8)	N26—N25—C33—C35	−179.0 (9)
N3—C2—C3—N1	3.3 (14)	N27—C32—C33—N25	−4.5 (16)
C4—C2—C3—N1	−176.4 (9)	C34—C32—C33—N25	175.2 (10)
N3—C2—C3—C5	−178.7 (9)	N27—C32—C33—C35	178.1 (10)
C4—C2—C3—C5	1.6 (14)	C34—C32—C33—C35	−2.2 (17)
N5—N6—C6—N8	177.8 (7)	C37—N31—C36—N32	178.3 (11)
Ag1—N6—C6—N8	4.4 (12)	C37—N31—C36—N30	−4.8 (17)
N5—N6—C6—N7	0.6 (12)	N29—N30—C36—N32	179.7 (10)
Ag1—N6—C6—N7	−172.8 (6)	N29—N30—C36—N31	2.9 (17)
C7—N7—C6—N8	−179.7 (7)	C36—N31—C37—C38	3.4 (18)
C7—N7—C6—N6	−2.4 (12)	C36—N31—C37—C39	−178.3 (10)
C6—N7—C7—C8	2.8 (12)	N30—N29—C38—C37	−1.7 (19)
C6—N7—C7—C9	−179.7 (8)	N30—N29—C38—C40	−179.5 (12)
N6—N5—C8—C7	−0.4 (12)	N31—C37—C38—N29	0(2)
N6—N5—C8—C10	179.8 (7)	C39—C37—C38—N29	−178.6 (12)
N7—C7—C8—N5	−1.5 (13)	N31—C37—C38—C40	177.4 (13)
C9—C7—C8—N5	−179.0 (8)	C39—C37—C38—C40	−1(2)
N7—C7—C8—C10	178.3 (8)	O2—S1—C41—F2	72.2 (15)
C9—C7—C8—C10	0.8 (14)	O1—S1—C41—F2	−46.1 (16)
C12—N11—C11—N12	−180.0 (9)	O3—S1—C41—F2	−168.7 (14)
C12—N11—C11—N10	0.5 (14)	O2—S1—C41—F1	−58.3 (14)
N9—N10—C11—N12	178.8 (8)	O1—S1—C41—F1	−176.6 (12)
Ag1—N10—C11—N12	3.8 (12)	O3—S1—C41—F1	60.9 (14)
N9—N10—C11—N11	−1.7 (13)	O2—S1—C41—F3	−174.1 (9)
Ag1—N10—C11—N11	−176.6 (6)	O1—S1—C41—F3	67.6 (11)
C11—N11—C12—C13	2.1 (14)	O3—S1—C41—F3	−54.9 (11)
C11—N11—C12—C14	−176.9 (8)	O4—S2—C42—F4	38 (2)
N10—N9—C13—C12	2.3 (13)	O6—S2—C42—F4	175.6 (16)
N10—N9—C13—C15	179.3 (8)	O5—S2—C42—F4	−70.1 (17)
N11—C12—C13—N9	−3.6 (15)	O4—S2—C42—F5	176.9 (15)
C14—C12—C13—N9	175.4 (9)	O6—S2—C42—F5	−45.9 (18)
N11—C12—C13—C15	179.6 (9)	O5—S2—C42—F5	68.4 (17)
C14—C12—C13—C15	−1.4 (15)	O4—S2—C42—F6	−74.1 (13)
C17—N15—C16—N14	−0.2 (14)	O6—S2—C42—F6	63.0 (12)
C17—N15—C16—N16	178.6 (9)	O5—S2—C42—F6	177.3 (11)

### Hydrogen-bond geometry (Å, °)

**Table:**

*D*—H···*A*	*D*—H	H···*A*	*D*···*A*	*D*—H···*A*
N4—H4A···N5	0.86	2.15	2.977 (9)	161
N8—H8A···N9	0.86	2.22	3.077 (10)	173
N12—H12B···N1	0.86	2.17	3.018 (11)	169
N16—H16B···N17	0.86	2.21	3.052 (10)	165
N20—H20A···N21	0.86	2.18	3.004 (9)	161
N24—H24B···N13	0.86	2.19	3.044 (11)	169
N4—H4B···N30^i^	0.86	2.10	2.935 (11)	162
N8—H8B···O1^ii^	0.86	2.19	3.032 (11)	168
N12—H12A···O6^iii^	0.86	2.26	3.047 (11)	152
N16—H16A···O3^iv^	0.86	2.23	3.032 (11)	155
N20—H20B···N26^ii^	0.86	2.15	3.003 (9)	172
N24—H24A···O4	0.86	2.26	3.121 (12)	175
N28—H28A···N19^ii^	0.86	2.24	3.084 (10)	167
N28—H28B···O5	0.86	2.22	2.978 (15)	146
N32—H32A···O2^ii^	0.86	2.17	2.982 (13)	156
N32—H32B···N3^i^	0.86	2.30	3.142 (12)	166
C5—H5B···O4	0.96	2.56	3.486 (17)	161
C9—H9A···N27^v^	0.96	2.56	3.363 (12)	141
C15—H15B···O3^iv^	0.96	2.58	3.538 (15)	177
C35—H35C···F1^iv^	0.96	2.54	3.375 (14)	145
C40—H40A···F5^iii^	0.96	2.43	3.208 (17)	138

Symmetry codes: (i) −*x*+2, −*y*+1, −*z*+1; (ii) −*x*+1, −*y*+1, −*z*+1; (iii) −*x*+2, −*y*+1, −*z*; (iv) *x*, *y*+1, *z*; (v) *x*, *y*, *z*+1.

## References

[bb1] Bruker (2007). *SMART*, *SAINT-Plus* and *SADABS* Bruker AXS Inc., Madison, Wisconsin, USA.

[bb2] Effendy, Marchetti, F., Pettinari, C., Pettinari, R., Skelton, B. W. & White, A. H. (2007). *Inorg. Chim. Acta*, **360**, 1451–1465.

[bb3] Jiang, Y.-H., Cui, L.-N., Huang, X., Jin, Q.-H. & Zhang, C.-L. (2011). *Acta Cryst.* E**67**, m1499. 10.1107/S1600536811040670 PMC3246931 22219751

[bb4] Jin, Q. H., Hu, K. Y., Song, L. L., Wang, R., Zhang, C. L., Zuo, X. & Lu, X. M. (2010*a*). *Polyhedron*, **29**, 441–445.

[bb5] Jin, Q. H., Song, L. L., Hu, K. Y., Zhou, L. L., Zhang, Y. Y. & Wang, R. (2010*b*). *Inorg. Chem. Commun.* **13**, 62–65.

[bb6] Liu, J. C., Guo, G. C., Ma, H. W., Yang, C., Zhou, G. W., Zheng, F. K., Lin, S. H., Wang, M. S. & Huang, J. S. (2002). *Chin. J. Struct. Chem.* pp. 371–373.

[bb7] Sang, R. L. & Xu, L. (2006). *Eur. J. Inorg. Chem.* pp. 1260–1267.

[bb8] Self, M. F., Pennington, W. T. & Robinson, G. H. (1991). *J. Coord. Chem.* **24**, 69–76.

[bb9] Sheldrick, G. M. (2008). *Acta Cryst.* A**64**, 112–122. 10.1107/S0108767307043930 18156677

[bb10] Wang, Y. & Cheng, P. (2007). *Struct. Chem.* pp. 667–682.

